# What Is Possible for Patients After Lung Transplantation? The Highest Reported Altitude Achieved by a Lung Transplant Recipient Without Supplemental Oxygen - Climbing Mount Aconcagua (6.961m)

**DOI:** 10.3389/ti.2026.16591

**Published:** 2026-05-12

**Authors:** Jakob Mühlbacher, Alexis Slama, Konrad Hoetzenecker, Christina Jelly, Holger Flick, Fedja Dzubur, Matthias P. Hilty, Paul Fellinger, Rodrigo Duplessis, Lukas Furtenbach, Ida Valerie Wedenig, Wilfried Wisser, Clemens Aigner, Peter Jaksch

**Affiliations:** 1 Department of Surgery, Division of Visceral Surgery, Medical University of Vienna, Vienna, Austria; 2 Department of Thoracic Surgery, Comprehensive Center for Chest Diseases, Medical University of Vienna, Vienna, Austria, United States; 3 Vanderbilt Lung Transplant, Vanderbilt Thoracic Surgery, Vanderbilt University Medical Center, Nashville, TN, United States; 4 Clinical Department of Pulmonology, Medical University of Graz, Graz, Austria; 5 Clinic for Respiratory Diseases Jordanovac, University Hospital Centre Zagreb, Zagreb, Croatia; 6 Institute of Intensive Care Medicine, University Hospital Zurich, Zurich, Switzerland; 7 Faculty of Medicine, University of Zurich, Zurich, Switzerland; 8 Department of Medicine III, Medical University of Vienna, Vienna, Austria; 9 Extreme Medicine, Medical Service of Aconcagua Provincial Park, Mendoza, Argentina; 10 Furtenbach Adventures, Innsbruck, Austria; 11 Department of Cardiac and Thoracic Aortic Surgery, Medical University of Vienna, Vienna, Austria

**Keywords:** extreme environments, high altitude, hypoxia, lung transplant, physical activity

Dear Editors,

Lung transplantation (LuTX) is an established and effective therapeutic option for patients with end-stage lung disease [1]. Over the past decades, advances in surgical techniques, perioperative management, immunosuppressive strategies, and long-term follow-up care have resulted in significant improvements in survival rates, health-related quality of life, and functional capacity after lung transplantation [2]. Consequently, many recipients are able to resume a broad range of physical activities, including high-intensity and endurance sports. Early publications on this topic are available from other solid organ transplant recipients [3]. In this context, participation in high-altitude mountaineering has been documented in carefully selected liver transplant recipients under close medical supervision [4]. In 2015 a transplanted patient reached the highest mountain peak (6.189m, Island Peak, Nepal) ever [5]. Also, lung transplant recipients are able to adapt to altitude and capable of performing prolonged exercise at high altitude after slow ascent [6, 7]. In 2017 eight lung transplanted patients successfully summited Mount Kilimanjaro (5.895 m, Tanzania) under guidance of the Vienna lung transplant team [7, 8]. Available evidence suggests that transplanted lungs retain the capacity to physiologically adapt to hypobaric hypoxia and can sustain prolonged physical exertion at high altitude, provided that ascent is gradual and appropriate acclimatization is ensured [7]. Eleven lung transplant recipients reached the summit of Mount Jebel Toubkal (4.167 m, Morocco) in 2019 without any adverse events, despite poorer cardiopulmonary performance compared to healthy volunteers [9]. In addition, they show stable immunosuppressive drug trough levels and stable Torque Teno virus loads suggest good immunologic tolerance relative to physical stress (Mühlbacher, accepted for publication in Scientific Reports, April 2026) [10].

As part of an international medical expedition under guidance of the Vienna lung transplant team and the respective national team leaders, nine transplanted patients (8 patients after lung transplantation, one patient after liver transplantation) were included to climb Mount Aconcagua (6.961m, Argentina) in January 2026. The expedition was supported by an accompanying team of physicians and professional guides. The actual tour planning was carried out by a professional expedition provider (Furtenbach Adventures GmbH, Rum, Austria) in cooperation with a local expedition provider (Grajales Expeditions, Los Penitentes, Mendoza Province), both of whom have many years of experience in planning and safely conducting expeditions. The selection of possible candidates was based on lung function and spiroergometry and was done in accordance with the included transplant centers in Austria, Switzerland, Croatia, Denmark and the USA.

Hypoxic conditioning (HC) applied at home as a pre-acclimatization strategy prior to high-altitude exposure may facilitate high-altitude ascents with a reduced risk of developing acute mountain sickness (AMS) [11, 12]. However, standardized protocols remain insufficiently defined, and robust scientific data are limited, although pre-acclimatization appears to be a key determinant in the success of rapid ascent expeditions [13]. It is currently unknown how this form of pre-acclimatization affects patients after lung transplantation. To maximize participant safety during the expedition, however, all participants completed a structured home-based HC program comprising at least 200 h of exposure prior to departure [11]. Participants completed mandatory safety and first aid training prior to the expedition; high flow oxygen systems (Summit Elite System, Summit Oxygen International Ltd) and carbon oxygen cylinders (4L, working pressure 300 bar; Armotech, Czech Republic) where available throughout the expedition for safety reasons.

The expedition to Mount Aconcagua (6.961 m, Argentina), followed a structured 19-day schedule organized by experienced professional providers. Accordingly, the ascent followed a standard acclimatization protocol *via* the normal route: approach to Plaza de Mulas Base Camp (BC) (4.350 m) over 4 days, followed by progressive establishment of higher camps at Plaza Canadá (5.050 m), Nido de Cóndores (5.560 m), and Camp Cólera (6.080 m). The summit attempt (6.961 m) was performed from High Camp Cólera (Figure 1A). Additional days were reserved for weather contingency and descent. Physiological monitoring, including heart rate and peripheral oxygen saturation, was performed using wearable devices provided within the framework of the project. In addition, the Lake Louise Acute Mountain Sickness (AMS) score was assessed daily based on self-reported symptoms documented by the mountaineers [14].

**FIGURE 1:**
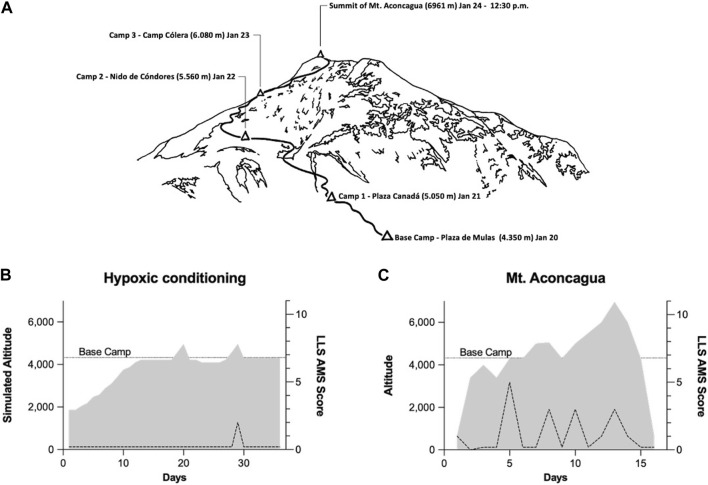
Schedule and route for the Mount Aconcagua expedition, times are given in local time **(A)**. Hypoxic pre-conditioning performed at home, illustrating the progression of simulated altitude exposure over time and the corresponding Lake Louise Score (LLS) for acute mountain sickness (AMS) **(B)**. Elevation profile during the Aconcagua expedition illustrating altitude exposure over time together with the corresponding AMS score **(C)**, all altitudes are reported in meters above sea level (m a.s.l.).

The majority of transplanted participants (7 patients after lung transplantation, one patient after liver transplantation) were unable to reach the summit due to a combination of altitude-related symptoms, reduced tolerance to physical demands at higher altitude, and precautionary decisions based on safety considerations, resulting in descent at various stages between base camp and the higher camps.

On January 24, a lung transplant recipient successfully reached the summit of Mount Aconcagua (6,961 m, Argentina) together with the accompanying expedition team (8 participants), all without the use of supplemental oxygen. The ascent and descent were completed without adverse clinical events. In particular, no signs or symptoms consistent with high-altitude pulmonary edema (HAPE) or high-altitude cerebral edema (HACE) were observed during high-altitude exposure.

This 51-year-old male lung transplant recipient (BMI 18.3 kg/m^2^), transplanted in 2002 for cystic fibrosis, resided at 407 m above sea level. Relevant comorbidities included diabetes mellitus and chronic kidney disease (creatinine: 2.57 mg/dL, November 2025); maintenance immunosuppression consisted of once-daily 0.75 mg extended-release tacrolimus in combination with everolimus 0.5 mg twice daily. He had prior high-altitude exposure, including Mount Kilimanjaro (5,895 m, Tanzania) [7], without any history of AMS, HAPE, or HACE. Baseline functional assessment demonstrated a maximal oxygen uptake (VO_2_max) of 30.9 mL·kg^-1^·min^-1^ (90% predicted; maximal workload 140 W) and an FEV_1_ of 2.7 L (86% predicted), indicating preserved exercise capacity and stable graft function prior to the expedition. As part of the pre-acclimatization strategy, the lung transplant recipient completed 311 h of HC over 36 days (Figure 1B). Following HC, hemoglobin levels remained stable (from 13.7 to 13.8 g/dL). As simulated altitude increased during the pre-acclimatization phase, the participant experienced only mild symptoms, which was accompanied by a corresponding elevation in AMS scores (maximum AMS-Score of 2, Figure 1B). On the mountain he reported only mild to moderate symptoms of AMS (maximum AMS-Score of 5), and no instances of HAPE or HACE occurred at any point during the expedition (Figure 1C). For mild gastrointestinal problems, he took one tablet of Metoclopramide (10 mg) daily for 5 days as the only additional medication during the expedition. Arterial PaO_2_ decreased by approximately 50%, from 82 mmHg in Mendoza (760 m) to 42 mmHg at Plaza de Mulas Base Camp (4,350 m), while arterial pH remained stable. Resting SpO_2_ values progressively declined with increasing altitude from of 89% at BC to 75% at Camp 3. In contrast, heart rate remained relatively stable throughout the stay on the mountain, with average values ranging from 90 bpm at BC to 95 bpm at Camp 3.

These findings should be interpreted cautiously, as such high-altitude performance is likely restricted to a highly selected subgroup of transplant recipients under exceptional medical supervision and is not generalizable to the broader transplant population. Although the observed VO_2_max of 31 mL·kg^-1^·min^-1^ is lower than values suggested in the literature for successful high-altitude mountaineering [15, 16], summit attainment in this case may potentially be explained by a combination of factors, including HC, a very slow ascent rate, minimal additional load supported by porter assistance, favorable environmental conditions and potentially also by psychological factors such as resilience and motivation in this highly selected patient population. Currently, evidence regarding VO_2_max thresholds for lung transplant recipients at high altitude remains limited.

In summary, selected patients after lung transplantation are able to tolerate and physiologically adapt to high-altitude exposure when preceded by normobaric hypoxic pre-acclimatization, without experiencing severe high-altitude–associated complications. Moreover, one lung transplant recipient successfully summited Mount Aconcagua without supplemental oxygen, which, to our knowledge, constitutes the highest reported altitude reached following lung transplantation.

## Data Availability

Raw data are available from the authors upon request.
